# Effects of stigma, hope and social support on quality of life among Chinese patients diagnosed with oral cancer: a cross-sectional study

**DOI:** 10.1186/s12955-020-01353-9

**Published:** 2020-04-28

**Authors:** Ying Zhang, Chunying Cui, Yu Wang, Lie Wang

**Affiliations:** Department of Social Medicine, School of Public Health, China Medical University, No.77 Puhe Road, Shenyang North New District, Shenyang, Liaoning 110122 People’s Republic of China

**Keywords:** Oral cancer, Stigma, Hope, Social support, Quality of life

## Abstract

**Background:**

Improving quality of life (QoL) has been one of the goals of health care for people living with oral cancer. This study aimed to assess QoL and investigate the effects of stigma, hope, and social support on QoL among Chinese oral cancer patients.

**Methods:**

A cross-sectional study was conducted at the Department of Stomatology, Shengjing Hospital of China Medical University and Stomatology Hospital of China Medical University in Liaoning Province, China, between May 2016 and October 2017. A total of 230 oral cancer patients were recruited to complete a questionnaire including the Functional Assessment of Cancer Therapy-Head and Neck (FACT-H&N), the Social Impact Scale (SIS), the Herth Hope Index (HHI) and the Multidimensional Scale of Perceived Social Support (MSPSS). Univariate one-way ANOVA/t-test, Person’s r and hierarchical linear regression analysis were conducted to explore the factors influencing QoL and the relationships between stigma, hope, perceived social support and QoL.

**Results:**

The mean QoL score was 90.85 ± 20.15 among the patients with oral cancer. Stigma was negatively related to QoL, explaining 39.3% of the variance. In addition, hope and perceived social support were positively associated with QoL, explaining 8.1% of the variance.

**Conclusion:**

Overall, Chinese patients with oral cancer suffer from low QoL. Stigma was significantly and negatively associated with QoL, while hope and perceived social support were positively associated with QoL. Oral cancer patients’ psychological states should be addressed, and adequate intervention based on positive psychological resources should be provided to improve the QoL of patients with oral cancer.

## Introduction

Oral cancer is a broad term for malignant tumours in the oral cavity, which may arise as a primary lesion in any part of the oral cavity or oropharynx, such as the lip, cheek, gingiva, tongue and floor of the mouth [1]. According to the World Health Organization (WHO), there were approximately 350,000 new cases and 170,000 deaths of oral cancer worldwide in 2018 [2]. However, it is estimated by the National Central Cancer Registry of China (NCCRC) that in 2014, the morbidity of oral cancer in China was 1.56/100,000, and the standardized morbidity of the world population was 0.99/100,000 [3]. In addition, oral cancer death accounted for approximately 1.13% of all cancer deaths in China in 2014 [3]. Although the incidence and mortality of oral cancer in China are not high, much attention should be directed to oral cancer because of the large population.

Quality of life (QoL) for oral cancer patients is a reflection of patients’ life status after this disease diagnosis. In addition, QoL has also become a significant factor in monitoring treatment and therapeutic procedure success in recent decades [4]. Poor QoL of oral cancer patients was has consistently been associated with longer hospital stays, poor postoperative performance, increased narcotic use and complications with treatment, and decreased treatment compliance [5]. Therefore, researchers are increasingly aware of the value of considering how to improve the level of QoL and extend survival length in patients with oral cancer [6]. Based on the literature review, we found that not only the general impacts of clinical and demographic differences but also psychological factors influence QoL.

In fact, several studies have reported on the prevalence of cancer stigma [7]. It has been found to be associated with various types of illness, including but not limited to HIV/AIDS, tuberculosis, and cancers [8]. Cancer is recognized as a stigma when it conveys deviation from the normal state. Once individuals are stigmatized, they are often stripped of their social status and become stereotyped, discriminated, and isolated or even feel exclusion from society [9]. Compared with patients with other types of cancer, patients with oral cancer always face substantial challenges, including problems with speaking, eating, breathing and facial disfigurement, which may significantly contribute to patient stigma. Stigma in cancer patients was proven to be strongly and consistently associated with negative psychological health, including depressive symptoms [10], anxiety [11], and demoralization [12]. Therefore, patients with oral cancer may experience more stigma that can result in poor psychological conditions and significantly impact their QoL.

In the past decade, positive psychological resources such as hope and perceived social support have become increasingly important in clinical practice. During treatment, hope can be regarded as one of the most important and effective coping styles when patients face cancer. A systematic review reported that hope was associated with better quality of life and spiritual well-being in Asian patients with cancer [13]. In addition, higher levels of social support lead individuals to believe that others are concerned about and accepting of them [14]. Many previous studies have demonstrated that social support is related to cancer patients’ QoL [15, 16]. Thus, we hypothesize that the presence of hope and supportive interpersonal relationships has the potential to influence the QoL of oral cancer patients in the present study.

However, to the best of our knowledge, extant studies have not yet explored the relationship of patients’ QoL with stigma, hope and social support in China. Therefore, in the current study, we aimed to assess QoL and the association of QoL with stigma, hope and social support in patients with oral cancer.

## Methods

### Study design and sampling

A cross-sectional study was carried out from May 2016 to October 2017. All participants were recruited from the Department of Stomatology of Shengjing Hospital of China Medical University and Stomatology Hospital of China Medical University. Once patients agreed to participate in this cross-sectional study, they were distributed questionnaires and an informed consent form to read and sign. Patients were included if they met the following criteria: (1) were at least 18 years old, (2) were diagnosed with oral cancers, (3) were post-surgery, (4) were aware of their own diagnosis, (5) were well enough to answer the questionnaires, and (6) were able to accurately and fluently answer questions. Patients with the following criteria were excluded: (1) had a history of psychiatric or cognitive disorders, (2) were illiterate and unable to complete the survey, and (3) had other oral diseases or other cancers. Investigators were responsible for helping them to read questionnaire items and providing explanation without any inducement. Of the 271 patients who met the inclusion criteria, 41 patients were excluded because the missing values exceeded 30% in the questionnaire. Finally, completed questionnaires were obtained from 230 individuals, including 135 males and 95 females. The effective response rate was 84.8%.

The study was approved by the Committee on Human Experimentation of China Medical University, and the study procedures were in accordance with ethical standards.

### Measurement of demographic and clinical variables

Demographic characteristics consisted of age, gender, marital status, education level, monthly income, work status, and residence. Clinical characteristics included the type of treatment, mandibulectomy (resection of the mandible), distant cancer metastasis, and familial inheritance (family history of cancer).

### Measurement of QoL

In this study, QoL was measured by the Functional Assessment of Cancer Therapy-Head and Neck (FACT-H&N), which comprises 39 questions and consists of five dimensions: physical well-being, social/familial well-being, emotional well-being, functional well-being, and head and neck cancer-specific subscales. Each item was rated on a five-point Likert scale from 0 for “not at all” to 4 for “very much”. The total score ranges from 0 to 144, with a higher score indicating a higher level of QoL. FACT-H&N as a specific instrument has been translated into many languages and validated in previous studies, including with Chinese patients [17–20]. In our study, Cronbach’s alpha coefficient for the scale was 0.931.

### Measurement of stigma

The Social Impact Scale (SIS), developed to assess the level of stigmatization for individuals with cancer or HIV/AIDS [21], has been applied to different populations with good reliability [22]. The 24-item SIS examined 4 domains of stigma: social rejection, financial insecurity, internalized shame, and social isolation. Responses to the scale were given on a 4-point Likert-type scale. Cronbach’s α coefficient for the scale was 0.947 in this study.

### Measurement of hope

The Herth Hope Index (HHI) [23] was used to assess hope in clinical patients with 12 items, and each item had 4 response categories from 1 to 4. The total score ranges from 12 to 48, with a higher total score reflecting a higher level of hope. The Chinese version of the HHI has demonstrated reliability and validity in cancer patients [24]. Cronbach’s α was 0.809 in the sample group.

### Measurement of social support

Social support was assessed using the 12-item version of the Multidimensional Scale of Perceived Social Support (MSPSS), which was developed by Zimet et al. [25]. This scale measured perceived social support from three domains: support from family, support from friends and support from significant others. The item has a 7-point rating ranging from “very strongly disagree” to “very strongly agree”. A higher total score reflects stronger social support. The Chinese version of the MSPSS was shown to have adequate reliability and validity for other cancer patients [26]. Cronbach’s alpha coefficient was 0.928 in the present research.

### Statistical analyses

This study used one-way ANOVA/t-test to describe the mean scores of QoL for different categorical demographic and clinical variables. Pearson’s correlation analysis was used to analyse the correlation among QoL, stigma, hope, and perceived social support. Hierarchical multiple regression analysis was conducted to investigate the effects of influencing factors on QoL. In hierarchical regression analysis, gender, age and potential variables (which were associated with QoL in univariate analysis) were entered in step 1. Stigma was added in step 2. Finally, the independent variables hope and social support were entered in step 3. The indicators provided in the regression models included *R*^2^, adjusted *R*^2^, *R*^2^ change, F-value and standardization regression coefficient (*β*). SPSS for Windows (version 22.0) was used to perform the statistical analyses, with a two-tailed *P*-value of < 0.05 considered to be statistically significant.

## Results

### Descriptive statistics

Demographic and clinical characteristics of the patients and the FACT-H&N scores for different categories of variables are shown in Table 1. Of the 230 patients, 135 (58.69%) were men, and 95 (41.31%) were women. The age of the patients ranged from 18 to 92 years, and the mean (SD) age was 56.13 (13.54) years old. A total of 89.13% of the participants were married or cohabiting, and 145 (63%) had a household monthly income under 3000 yuan. With regard to clinical variables, only 52 (22.6%) of the patients had undertaken reparative therapy, and 182 (79.1%) of the patients used marginal mandibulectomy. Of all the variables, the FACT-H&N scores were significantly different among the different categories of variables, including gender (*t* = 7.060, *p* = 0.008), marital status (*t* = 6.496, *p* = 0.011), residence (*t* = 5.119, *p* = 0.025), educational background (*F* = 3.527, *p* = 0.029), mandibulectomy (*t* = 2.644, *p* = 0.009), and distant metastasis (*t* = 2.951, *p* = 0.003); however, the differences in the other variables were not statistically significant.

**Table 1:** Demographic and clinical characteristics and the score of QoL among oral cancer patients

Variables	N (%)	FACT-H&N			
		Mean	SD	*F*/*t*	*P*-value
Gender				7.060	0.008^**^
male	135 (58.6)	87.93	19.03		
female	95 (41.4)	95.01	21.05		
Age				1.289	0.199
< 60	139 (60.4)	92.42	22.32		
≥ 60	91 (39.6)	88.99	17.13		
Marital status				6.496	0.011^**^
Single/divorced/Separated/widow	25 (10.8)	99.44	19.07		
Married/cohabitation	205 (89.2)	89.68	20.01		
Residence				5.119	0.025^*^
Rural area	145 (63.1)	93.13	19.42		
Urban area	85 (36.9)	86.96	20.87		
Educational background				3.597	0.029^*^
Primary/middle school	101 (43.9)	90.40	17.36		
High / secondary/junior school	102 (44.3)	88.80	21.41		
College or above	27 (11.7)	100.29	22.93		
Household monthly income (yuan)				1.637	0.202
< 3000	145 (63.0)	89.93	20.58		
≥ 3000	85 (37.0)	95.62	22.02		
Job status				1.353	0.261
Retirement/unemployed	133 (57.8)	91.31	18.71		
Temporary workers	32 (13.9)	85.48	20.42		
Regular employee	65 (28.2)	93.50	22.69		
Reparative therapy				1.321	0.188
Yes	52 (22.6)	87.61	16.92		
No	178 (77.4)	91.80	20.94		
Mandibulectomy				2.644	0.009^***^
Yes	182 (79.1)	92.63	19.93		
No	48 (20.9)	84.14	19.74		
Distant metastasis				2.951	0.003^***^
Yes	12 (5.2)	74.41	22.08		
No	218 (94.8)	91.76	19.69		
Familial inheritance				1.009	0.314
Yes	15 (6.5)	95.93	15.07		
No	215 (93.5)	90.93	20.43		

One-way ANOVA / t-test were used to describe the mean scores of QoL in different categorical demographic and clinical variables

*SD* standard deviations

^*^p < 0.05, ^**^p < 0.01, ^***^p < 0.001

### Correlation between continuous resources and QoL

The results of correlation analyses between the continuous variables are presented in Table 2. The mean QoL score among oral cancer patients was 90.85 ± 20.15. FACT-H&N scores were negatively and significantly correlated with stigma (*r* = −0.700, *p* < 0.01) and positively and significantly correlated with hope (*r* = 0.415, *p*<0.01) and perceived social support (*r* = 0.526, *p* < 0.01).

**Table 2:** Scores and correlation of FACT-H&N with other variables

	Mean	SD	FACT-H&N	Stigma	Hope	Social support
FACT-H&N	90.85	20.151	1			
Stigma	73.47	12.550	−0.700^**^	1		
Hope	34.72	3.822	0.418^**^	−0.311^**^	1	
Social support	60.20	11.334	0.550^**^	−0.393^**^	0.563^**^	1

Pearson’s correlation analysis was used to analyze the correlation among QoL, stigma, hope, perceived social support

*SD* standard deviations

^**^p < 0.01

### Hierarchical multiple regression analysis

As shown in Table 3, all the independent variables that were associated with oral cancer patients’ QoL in univariate analysis (*p* < 0.05) were entered into the hierarchical multiple regression model. Each step of independent variables made a significant contribution to the variance in QoL. In step 1, demographic characteristics, including gender, marital status, residence area, and education level, and clinical variables, including distant metastasis and mandibulectomy as a whole, accounted for 9.9% of the variance in QoL. In step 2, after controlling for demographic and clinical characteristics, this study found stigma to be significantly and negatively associated with QoL. Stigma exerted a significant effect on the QoL of oral cancer patients (*F* = 26.855, adjusted *R*^2^ = 0.504, *R*^2^ change = 0.393). In step 3, positive psychological resources, including hope and perceived social support, were added into the model, and they were significantly positively associated with QoL. The two independent variables accounted for 8.1% of the variance in QoL. In addition, all variables in the model could explain 58.5% of the variance in QoL.

**Table 3:** Hierarchical multiple regression analysis results of QoL

Variables	Step 1		Step 2		Step 3	
	*β*	*P*-value	*β*	*P-*value	*β*	*P*-value
Control variables						
gender	0.068	0.441	0.053	0.414	0.065	0.280
marriage	−0.110	0.090	−0.128^**^	0.008	−0.086	0.055
residence	− 0.176^*^	0.011	− 0.020	0.707	− 0.007	0.886
Mandibulectomy	−0.128^*^	0.048	− 0.049	0.312	−0.045	0.315
Distant metastasis	−0.160^**^	0.007	−0.037	0.445	0.029	0.552
Edu-1	0.052	0.449	−0.076	0.144	−0.059	0.218
Edu-2	0.004	0.954	−0.071	0.183	−0.076	0.116
Stigma			−0.681^***^	0.000	−0.571^***^	0.000
hope					0.117^*^	0.031
Social support					0.261^***^	0.000
*F*	4.145^***^		26.855^***^		30.327^***^	
*R*^*2*^	0.130		0.523		0.605	
Adjusted *R*^*2*^	0.099		0.504		0.585	
*R*^2^-change	0.130		0.393		0.081	

Hierarchical multiple regression analysis was conducted to investigate the effects of influence factors on QoL

Edu-1 means “primary/middle school” vs. “college or above”, Edu-2 means “high/secondary/junior school” vs. “high or secondary school”

^*^p < 0.05, ^**^p < 0.01, ^***^p < 0.001

## Discussion

This relatively large-scale cross-sectional study of patients with oral cancer was conducted in the northeastern region of China and was the first study to examine the effects of stigma, hope and social support on QoL in China. Our findings showed that the mean QoL score among Chinese patients with oral cancer was 90.85 ± 20.15, which is lower than that of patients with other head and neck cancers in China (such as nasopharyngeal carcinoma and laryngeal cancer patients) [27, 28]. In addition, Tulio et al. indicated that in Brazil, the mean FACT-H&N score was 96.6 ± 20.5 [29]. In Williams’ research, the median FACT-H&N score was 110.20 in America [30]. Dominika et al. reported that in Poland, the mean FACT-H&N score was 109.19 [31]. By contrast, the QoL of Chinese oral cancer patients in our study was low. Although there is continual progress in diagnosis and treatment technology and it is becoming increasingly convenient to obtain quality care in China, our results found that Chinese patients with oral cancer still suffered from poor QoL. Therefore, it is very important to identify the essential influencing factors and targeted solutions to improve their QoL.

Among all the demographic and clinical variables, some variables were related to QoL, including marital status, residence area, educational level, undergone mandibulectomy and distant metastasis. These factors in combination explained 9.9% of the variance in the QoL of patients with oral cancer. The findings of our study showed that patients with a college education or higher had a higher QoL score, which echoed the findings of Liu’s study [32]. On the one hand, higher education levels were proven to be positively related to high resilience [33], and resilient patients are always considered to possess stronger abilities to rebound from frustration and tragedy [34]. On the other hand, patients with higher education have more access enabling them to obtain disease-related knowledge and better understand their condition. This result indicated that more communication between medical workers and patients is essential for improving patient QoL. Moreover, as the results showed, patients with distant metastasis had the lowest QoL in this study. However, metastasis is one of the main causes of oral cancer patient death, which is the worst negative event among cancer patients [35]. In addition to oral cancer, metastasis has been confirmed as an adverse influential factor in many other cancers in several studies [36–38].

One of the core findings in this study was that stigma alone explained more than one-third of the variance in QoL. Specifically, stigma was found to be significantly negatively associated with QoL, which was consistent with previous studies [39, 40]. Treatment for oral cancer is complex, which can lead to functionality issues such as dysphagia and breathing difficulties, as well as a cosmetic burden with facial disfigurement. However, facial disfigurement is always associated with the development of shame and the perception of stigma [11]. The sense of inner shame plays a crucial role in these patients’ self-evaluation processes. High levels of stigma not only have a negative impact on follow-up treatments, including treatment compliance, treatment-seeking behaviours, self-esteem and social adaptation, but are also harmful to patients’ recovery, seriously impairing their QoL. Noticeably, the mean stigma score of the participants in this study was found to be higher than that of their counterparts in other countries [41–43], which may partly explain why Chinese patients with oral cancer experienced poor QoL. However, to our knowledge, there are no other published clinical interventions addressing stigma in oral cancer patients so far [42]. Therefore, future studies should focus more on positive psychological factors that may be relevant when stigma impacts oral cancer patients’ QoL.

In this study, perceived social support and hope were found to be positively associated with the QoL of patients with oral cancer. Patients with a high level of hope were likely to experience high QoL, which is consistent with a previous study [44]. Furthermore, a higher level of hope was confirmed to be associated with lower rates of alcohol and cigarette abuse, more frequent exercise and better nutrition, which are critical factors in preventing cancer recurrence and increasing QoL [45]. As can be expected, oral cancer patients with higher levels of hope experience higher QoL because they are more confident both in daily life and in disease states. Thus, strengthening hope is an important strategy to increase the QoL of patients with oral cancer in China. In addition, we found that social support was a predictive factor for QoL and was positively associated with QoL. A previous study showed that the absence of social support after diagnosis and during treatment was associated with the development of depression and anxiety and eventually affected the treatment effect [46]. Likewise, Hodges also indicated in their study that social support was positively related to patients’ QoL and promoted well-being, ultimately improving QoL [47]. Therefore, more social support from family and friends is essential to improve overall QoL.

The most important value of this study is that we identified that stigma was significantly negatively associated with QoL in oral cancer patients. In addition, our study has added to the evidence that the positive resources of hope and social support were positively associated with QoL in oral cancer patients. Based on our findings, some implications should be mentioned. First, Chinese medical institutions and government should pay more attention to oral cancer patients with low levels of QoL. Second, it is important for clinicians and nurses to pay more attention to patients with distant metastasis. Third, more attention should be devoted to patients with high stigma. Some studies have confirmed that contact with health professionals and the community [48], peer counselling [49], skill building and empowerment [50] were efficient for decreasing stigma. Thus, future research should focus more on longitudinal studies to determine whether reducing stigma helps improve QoL. Finally, health care organizations should recognize the importance of psychological strengths for oral cancer patients to combat severe diseases. More target intervention strategies should be conducted in future studies.

Nonetheless, there were some limitations in our study. First, because of the cross-sectional design and self-report measures, no conclusions could be drawn about a causal relationship between psychosocial resources and QoL. Second, this study was conducted in one city from a province of the northeastern region of China. The representativeness of the sample might thus be affected. Third, a control group should be included in our study. Thus, the interpretation of the results would be more convincing. Last, several potential factors, such as recurrence and pathological stage, may affect the QoL of patients with oral cancer, which we did not include in our study. A larger and longitudinal study should be conducted in future research.

## Conclusion

In conclusion, our study shows that patients with oral cancer in northeastern China suffer from relatively low QoL. Stigma was significantly and negatively associated with QoL, while hope and perceived social support were positively associated with QoL. Thus, in clinical practice, more attention should be devoted to patients’ psychological status, and more social support from family, friends or clinical workers should be provided to Chinese oral cancer patients. More importantly, more psychological intervention based on positive psychological resources should be introduced to improve QoL.

## Data Availability

The datasets analyzed during the current study are available from the corresponding author on reasonable request.

## Abbreviations

WHO: World Health Organization
NCCRC: National Central Cancer Registry of China
QoL: Quality of Life
FACT-H&N: the Functional Assessment of Cancer Therapy-Head and Neck
SIS: the Social Impact Scale
HHI: the Herth Hope Index
MSPSS: the Multidimensional Scale of Perceived Social Support
SD: Standard deviation
